# Immunopositivity for Histone MacroH2A1 Isoforms Marks Steatosis-Associated Hepatocellular Carcinoma

**DOI:** 10.1371/journal.pone.0054458

**Published:** 2013-01-23

**Authors:** Francesca Rappa, Azzura Greco, Christine Podrini, Francesco Cappello, Michelangelo Foti, Lucie Bourgoin, Marion Peyrou, Arianna Marino, Nunzia Scibetta, Roger Williams, Gianluigi Mazzoccoli, Massimo Federici, Valerio Pazienza, Manlio Vinciguerra

**Affiliations:** 1 Department of Experimental Biomedicine and Clinical Neurosciences, Section of Human Anatomy, University of Palermo, Palermo, Italy; 2 Euro-Mediterranean Institute of Science and Technology, Palermo, Italy; 3 Institute of Hepatology, Foundation for Liver Research, London, United Kingdom; 4 Istituto “Paolo Sotgiu, Libera Università degli Studi di Scienze Umane e Tecnologiche, Lugano, Switzerland; 5 Department of Cell Physiology and Metabolism, University of Geneva, Geneva, Switzerland; 6 Department of Systems Medicine, University of Rome Tor Vergata, Rome, Italy; 7 Pathologic Anatomy Unit, Civic Hospital, Palermo, Italy; 8 Department of Medical Sciences, Division of Internal Medicine and Chronobiology Unit, IRCCS “Casa Sollievo della Sofferenza” Hospital, San Giovanni Rotondo, Italy; 9 Division and Laboratory of Gastroenterology, IRCCS “Casa Sollievo della Sofferenza” Hospital, San Giovanni Rotondo, Italy; University of Texas Health Science Center at San Antonio, United States of America

## Abstract

**Background:**

Hepatocellular carcinoma (HCC) is one of the most common cancers worldwide. Prevention and risk reduction are important and the identification of specific biomarkers for early diagnosis of HCC represents an active field of research. Increasing evidence indicates that fat accumulation in the liver, defined as hepatosteatosis, is an independent and strong risk factor for developing an HCC. MacroH2A1, a histone protein generally associated with the repressed regions of chromosomes, is involved in hepatic lipid metabolism and is present in two alternative spliced isoforms, macroH2A1.1 and macroH2A1.2. These isoforms have been shown to predict lung and colon cancer recurrence but to our knowledge, their role in fatty-liver associated HCC has not been investigated previously.

**Methods:**

We examined macroH2A1.1 and macroH2A1.2 protein expression levels in the liver of two murine models of fat-associated HCC, the high fat diet/diethylnistrosamine (DEN) and the phosphatase and tensin homolog (PTEN) liver specific knock-out (KO) mouse, and in human liver samples of subjects with steatosis or HCC, using immunoblotting and immunohistochemistry.

**Results:**

Protein levels for both macroH2A1 isoforms were massively upregulated in HCC, whereas macroH2A1.2 was specifically upregulated in steatosis. In addition, examination of human liver samples showed a significant difference (p<0.01) in number of positive nuclei in HCC (100% of tumor cells positive for either macroH2A1.1 or macroH2A1.2), when compared to steatosis (<2% of hepatocytes positive for either isoform). The steatotic areas flanking the tumors were highly immunopositive for macroH2A1.1 and macroH2A1.2.

**Conclusions:**

These data obtained in mice and humans suggest that both macroH2A1 isoforms may play a role in HCC pathogenesis and moreover may be considered as novel diagnostic markers for human HCC.

## Introduction

The current pandemic in obesity/metabolic syndrome is a risk factor for many types of cancer. The largest increase in cancer risk (∼5 fold) in obese individuals with high body mass index (BMI 35–40) was seen for primary hepatocellular carcinoma (HCC) [1]. Obesity is accompanied in up to 90% of cases by non-alcoholic fatty liver disease (NAFLD) [2]. The latter is the consequence of an imbalance between lipid availability through fatty acid uptake and *de novo* lipogenesis, and lipid secretion and disposal via free fatty acid oxidation, resulting in hepatic accumulation of lipids (steatosis) [3]. In 10% of the cases NAFLD will progress to a steatohepatitis (NASH), and in 8–26% to cirrhosis, with an increasingly reported percentage of cases with cirrhosis or NAFLD at an earlier stage developing HCC [4]. Recent studies in rodent models suggested that high fat in the liver might trigger the development of HCC through inflammation, activating specific signalling pathways, growth factors and cytokines [5], [6], [7], [8], [9], [10].

Alterations in hepatocyte metabolism and proliferation during steatosis and HCC are triggered by changes in gene transcriptional patterns. The epigenetic mechanisms involved in HCC associated with obesity/metabolic syndrome/steatosis have not been investigated in detail. Nuclear chromatin compaction is regulated at several levels, allowing transcriptional plasticity [11]: one of these is the replacement of canonical histones around which DNA is wrapped (H2A, H2B, H3 and H4) with the incorporation of histone variants. The histone variant of histone H2A known as macroH2A1 is believed to act as a strong transcriptional modulator that can either repress transcription [12], [13], but can also activate a subset of genes in response to as yet undefined growth signals [14], [15]. MacroH2A1 knock out (KO) mice display hepatic steatosis and derangements in glucose and lipid metabolism [16], [17] and, interestingly, when wild type mice are fed a methyl-deficient diet, which induce a fatty liver and inflammation, a total increase in the hepatic content of macroH2A1 is observed [18].

MacroH2A1 is present in 2 isoforms, macroH2A1.1 and macroH2A1.2, which are generated upon RNA alternative exon splicing. The expression of both isoforms has been shown to predict lung cancer recurrence [19] and, in colon cancer, macroH2A1.1 inversely correlates with cancer severity and survival, whereas macroH2A1.2 does not show such correlation [20]. Recently, it has been shown that splicing of macroH2A1 isoforms regulates cancer cell growth [21]. In A549 lung cancer cells, HeLa cervical adenocarcinoma cells, IMR90 primary lung fibroblasts, and MG-63 osteosarcoma cells reduced levels of macroH2A1.1 compared to macroH2A1.2 were observed [21]. Reintroduction of macroH2A1.1 suppressed the growth of these cancer cell lines [21]. Other studies, which did not distinguish between the isoforms, demonstrated that KO of all macroH2A1 isoforms induced the progression of the melanoma malignant phenotype both *in vitro* and *in vivo* through increased expression of CDK8 oncogene [22].

Regulation by macroH2A1 of oncogenes and/or tumor suppressors’ expression in hepatocytes could be particularly relevant for fatty liver-associated HCC, since the activities of these genes often link mechanistically hepatic steatosis to the onset of HCC, as we have previously shown for tumor suppressor phosphatase with tensin homology (PTEN) [23], [24], [25]. PTEN is one of the most important tumor suppressors, mutated or deleted in nearly half of human cancers, including HCC patients [26] and changes in its expression have also been shown to regulate hepatic lipid metabolism and insulin sensitivity [23], [24], [25], [26]. In this study we explored if an altered expression of macroH2A1 isoforms might be associated to fatty liver-associated HCC using two robust mice models for this transition, the dietary high fat/diethynitrosamine (DEN) diet [8] and the genetic liver-specific PTEN knock-out (KO) mouse [27]. Furthermore, we examined the expression of macroH2A1 isoforms in human liver biopsies from patients where HCC occurred in a background of pure steatosis, in absence of other liver diseases.

## Materials and Methods

### Ethics Statement

Human biopsies: all the procedures followed were in accordance with the ethical standards of the responsible committees (institutional and national) on human experimentation and with the Helsinki Declaration of 1975 (as revised in 2008). Written informed consents were obtained from all patients at the time of biopsy and the study was approved by Ethics Committee of the Civic Hospital, Palermo, Italy.

Mice models: for the PTEN KO model, all the procedures were in accordance with the Swiss guidelines for animal experimentation and with ethically written approval by the Geneva (Switzerland) health head office. For the HF/DEN model, procedures were in accordance with the Italian national authorities and written approval was obtained by the Institutional Animal Care of the University of Rome "Tor Vergata” protocol 28/02/2012 n. 17.

### Mice Models

According to an established protocol [8], in the DEN-induced HCC model, DEN (25 mg/kg) was injected intraperitoneally into 14 days old mice. After 4 weeks, mice were separated into two dietary groups and fed either chow or high fat diet until sacrificed at 36 weeks of age. High fat diet (composed of 59%-fat, 15%-protein, 26%-carbohydrates based on caloric content) was purchased from Research Diet, New Brunswick, NJ, US.

To obtain PTEN liver specific KO mice, Pten^flox/flox^ mice (129Ola ∞ C57BL6/J F2) were mated to AlbCre transgenic mice (C57BL6/J background) as previously described [27], in which expression of Cre is controlled by the promoter of the hepatocyte-specific gene Albumin. Offspring carrying AlbCre and two copies of the floxed Pten allele (AlbCrePten^flox/flox^), and control Pten^flox/flox^ mice were retained for experiments. Mice were sacrificed at 16 weeks for studying the steatotic phenotype and at 52 weeks for studying the HCC phenotype. PCR analysis of PTEN genotypes was performed as previously reported [27].

In both high fat diet DEN-induced HCC and PTEN KO-induced HCC models, tumors in each liver lobe were counted. Serum, liver tumor and non-tumor tissue was collected and rapidly frozen for biochemical and histological analyses (see below).

### Human Sample Collection

Formalin-fixed paraffin embedded biopsies were retrospectively collected from files of the Pathologic Anatomy Unit of the Civico Hospital, Palermo, Italy. 10 cases were selected of mild mixed macro- and micro-vesicular steatosis. 10 cases of HCC arising in macro-vesicular steatosis were also selected. The clinical characteristics of the patients studied are summarized in Table 1, in terms of history of either HBV/HCV infection, cirrhosis, alcoholism and NAFLD score (see next paragraph). Fibrosis and/or cirrhosis were not observed in the biopsies.

**Table 1:** Clinical characteristics of the patients studied.

DISEASE	# OF CASES	SEX(M/F)	AGE RANGE (mean)	HBV	HCV	NAFLD SCORE* RANGE	CIRRHOSIS	ALCOHOLISM
STEATOSIS	10	5/5	45–71 (59.75)	0/10	0/10	0–2	0/10	0/6 (UNKNOWN IN 4 CASES)
HCC	10	5/5	40–75 (57.90)	0/10	0/10	0–4	0/10	0/10

* Assessed according to Ref. 29.

### Histological Assessment of NAFLD Score

Sections from both mice and human specimens were processed by haematoxylin and eosin (H&E) and Masson trichrome staining for histological evaluation of NAFLD score, as previously described [28], [29], in which a semi-quantitative scoring system that grouped histological features into five broad categories of steatosis, inflammation, hepatocellular injury, fibrosis, and miscellaneous features was performed.

### Western Blot Analyses

Cytoplasmic and nuclear protein extraction from nontumorous liver parenchyma and HCC tissue preparations and immunoblotting analyses were performed as previously described [23], [25]. Histone fraction was enriched using an acid extraction protocol. Briefly, The snap-frozen tissues were suspended and homogenized in 200 µl of H-lysis solution (0.2 M sucrose, 3 mM CaCl2, 1 mM Tris-HCl pH8.0, 0.5 NP40, protease inhibitor cocktail), incubated on ice for 8 min; centrifuged at 1.300×g, 4°C, for 5 min to separate supernatant from nuclei fraction (P1). P1 was washed once with H-wash solution (300 mM NaCl, 5 mg MgCl2, 5 mM DTT, 0.5% NP40)and lysed for 30 min in 100 µl H-extract solution (0.5 mM HCl, 10% glycerol, protease inhibitor cocktail), followed by centrifugation at 13.000×g 4°C, for 5 min. Finally, TCA precipitation was performed. Antibodies against histone H3 (Activ Motif) were use to normalize protein levels.

### Immunohistochemistry

Immunostainings were performed by iVIEW DAB Detection Kit for Ventana BenchMark XT automated slide stainer on human biopsies [30]. Primary antibodies for MacroH2A1.1 and MacroH2A1.2 were generated at the European Molecular Biology Laboratory (EMBL) and were a courtesy of Prof. Andreas Ladurner (Ludwig Maximilian University, LMU, Munich, Germany). Positivity for Ki-67 (Vector VP-K451, DBA ITALIA S.R.L., Milan, Italy ) was also examined. All primary antibodies were diluted 1∶100. Positive and negative controls were run concurrently. Immunopositive evaluations were performed in blind by three expert pathologists (FR, FC and NS) and percentage of positive nuclei (tumor cells in HCC and hepatocytes in steatosis) was calculated in ten random high power fields at a magnification of 400x.

### Statistical Analysis

Results are expressed as means ± S.E. Comparisons were made by using Student’s t test. Differences were considered as significant when P<0.05, P<0.01 or P<0.001, as indicated in the Figures and Figure Legends.

## Results

### MacroH2A1.1 and macroH2A1.2 Expression in the Liver of High Fat Diet fed/DEN Mice

**Figure 1:**
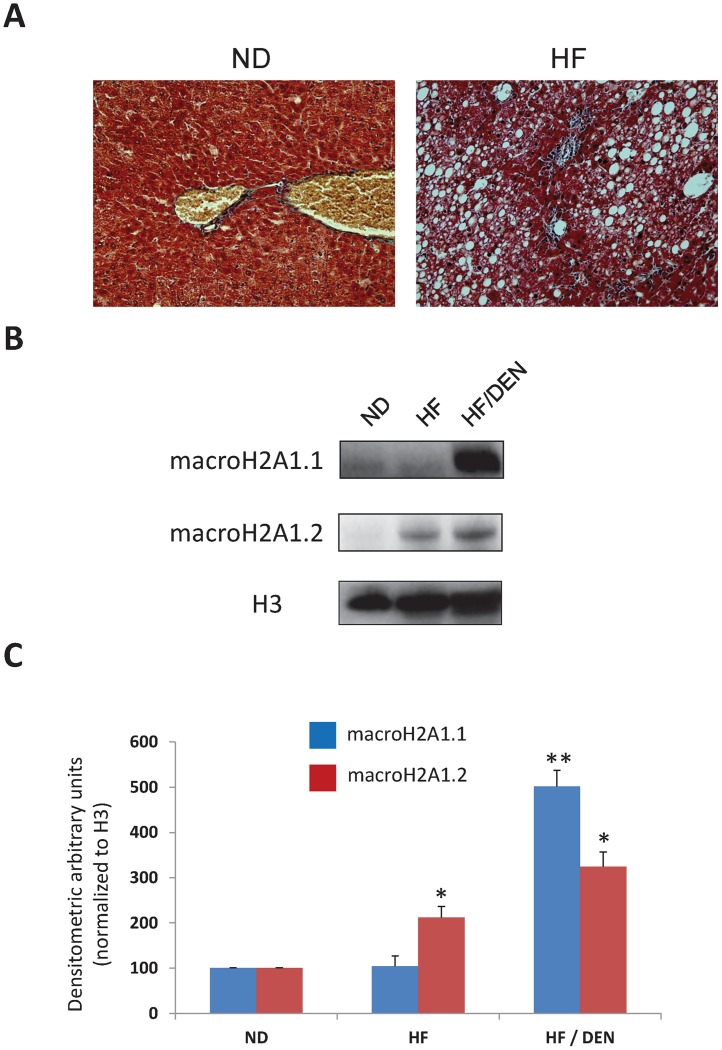
Protein expression of macroH2A1 isoforms in the liver of HF/DEN mouse model of steatosis and HCC. **A.** Representative pictures of trichrome staining in liver samples from mice fed a normal diet (ND) and mice fed a high fat (HF) diet for 36 weeks. **B.** Histone extracts from livers of ND, HF and HF/DEN mice were processed for immunoblotting. MacroH2A1.1, macroH2A1.2 and histone H3 (loading control) protein levels were detected with specific antibodies. Representative immunoblots are shown **C**. Densitometric quantification of macroH2A1.1, macroH2A1.2 protein levels in the livers of ND, HF and HF/DEN mice. N = 5, * P<0.05, ** P<0.01 versus ND mice.

A potent *bona fide* dietary mouse model of high fat-induced HCC developed recently was reproduced in this study [8]. Male mice maintained on HF gained more weight than mice on a normal diet (ND), developed glucose intolerance and their relative liver weight and triglycerides were increased (*data not shown*) [8]. This was accompanied by increased hepatic steatosis with a mean of NAFLD score of 1 *versus* 5, respectively (Fig. 1A). Mice fed with ND and treated with DEN injection at a low dose of 25 mg/kg did not display steatosis, they were indistinguishable from animal fed a ND (*data not shown*) and therefore they were not retained for further analyses. At sacrifice, mice injected with DEN and kept on HF exhibited HCC nodules, as well as augmented levels of inflammatory cytokines IL-6, TNFα and IL-1β mRNAs, while mice under ND did not (*data not shown*) [8].

To determine whether protein expression levels of macroH2A1.1 and macroH2A1.2 were altered in the context of steatosis or HCC, a histone extraction protocol was applied to the livers of mice fed a ND, fed with HF or injected with DEN and fed with HF (HF/DEN), followed by immunoblotting analysis. MacroH2A1.1 protein was found weakly expressed in the liver of ND or HF mice, while macroH2A1.2 expression was significantly increased in HF-fed mice compared to ND-fed mice (Fig. 1B). Both macroH2A1.1 and macroH2A1.2 expression levels were highly enhanced in the HCC tissue of HF/DEN treated animals (Fig. 1B). Thus, in the HF/DEN dietary model of steatosis and HCC, both macroH2A1 isoforms were associated with cancer, whereas macroH2A1.2 is specifically increased in the presence of fat.

### MacroH2A1.1 and macroH2A1.2 Expression in the Liver of Liver-specific PTEN KO Mice

**Figure 2:**
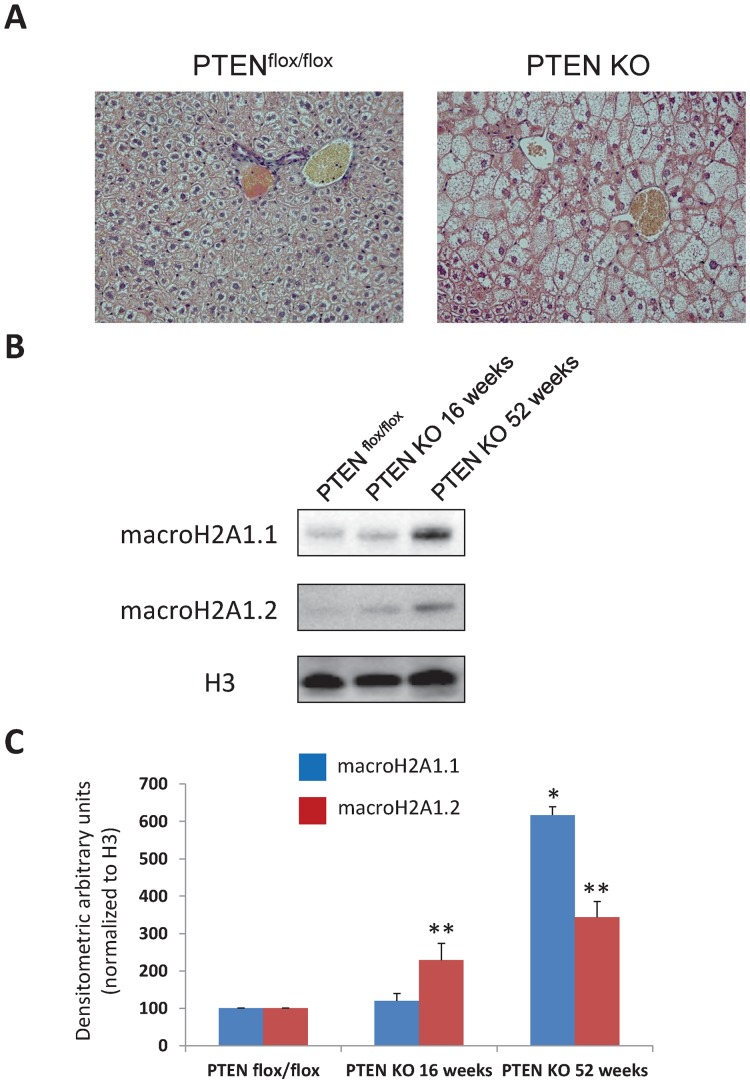
Protein expression of macroH2A1 isoforms in the liver of the liver-specific PTEN KO mouse model of steatosis and HCC. **A.** Representative pictures of trichrome staining in liver samples from PTEN^flox/flox^ and PTEN KO mice. **B.** histone extracts from livers of 16 weeks old PTEN^flox/flox^, 16 weeks old PTEN KO and from liver tumors of 52 weeks old PTEN KO mice were processed for immunoblotting. MacroH2A1.1, macroH2A1.2 and histone H3 (loading control) protein levels were detected with specific antibodies. Representative immunoblots are shown. **C.** Densitometric quantification of macroH2A1.1, macroH2A1.2 protein levels in the livers of 4 months old PTEN^flox/flox^ (N = 5), PTEN KO 16 weeks old (N = 5) and in the liver tumors of PTEN KO 52 weeks old mice (N = 2), * P<0.05, ** P<0.01 versus PTEN^flox/flox^ mice.

Hepatocyte-specific PTEN deficiency results in steatosis and HCC in mice, at 10–16 and 74–78 weeks of age, respectively [27]. Expression of adipogenic and lipogenic genes, such as PPARγ, is increased in the liver of hepatocyte-specific PTEN KO mice [27]. The transition from steatosis to HCC is accompanied in these animals by NASH and the appearance of liver adenomas at 40–44 weeks of age [27]. Paradoxically, PTEN being a negative regulator of insulin signalling [31], liver-specific PTEN KO mice displayed hepatic insulin hypersensitivity and increased systemic glucose tolerance [27]. We used this well established genetic model to study the expression of macroH2A1 isoforms in hepatic steatosis and HCC. PTEN^flox/flox^ mice were crossed to AlbCre transgenic mice, in which expression of Cre is controlled by the promoter of the hepatocyte-specific gene albumin. Control PTEN^flox/flox^ mice and PTEN KO obtained by AlbCre-mediated deletion of both PTEN alleles were retained for experiments. Animals were sacrificed at 16 and 52 weeks of age for histological and biochemical analyses. As expected the liver of 16 weeks old PTEN KO mice showed extensive fat accumulation compared to 16 weeks old PTEN^flox/flox^ littermates (mean NAFLD score 4 *versus* 1, respectively), (Figure 2A). We assessed if the protein expression levels of macroH2A1.1 and macroH2A1.2 were altered in the context of steatosis or HCC in liver specific PTEN KO mice. Immunoblotting analysis on histone extract revealed that macroH2A1.1 levels were low both in the liver of PTEN^flox/flox^ mice and of 16 weeks old PTEN KO, while macroH2A1.2 expression was greatly enhanced in the liver of 16 weeks old PTEN KO compared to age-matched PTEN^flox/flox^ mice (Fig. 2B). Both macroH2A1.1 and macroH2A1.2 protein expression levels were massively increased in the HCC tissue of 52 weeks old PTEN KO mice (Fig. 2B). Similarly to the HF/DEN model, in the PTEN KO model of steatosis and HCC both macroH2A1 isoforms associate with cancer, whereas macroH2A1.2 is specifically upregulated in the fatty liver.

### MacroH2A1.1 and macroH2A1.2 Expression in the Liver of Steatotic/HCC Patients

**Figure 3:**
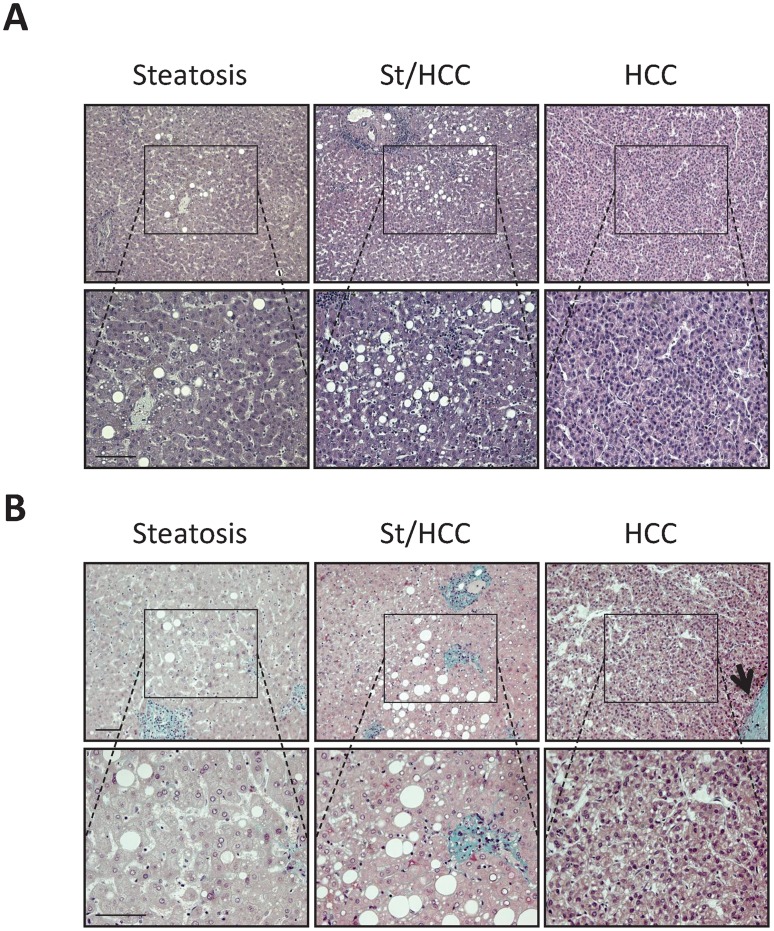
Histological features of liver biopsies of patients with steatosis and HCC. **A.** Representative pictures of hematoxylin and eosin (H/E) staining. In HCC samples both area with tumor and area of steatosis close to HCC (St/HCC) were examined. Pictures showed the same area observed with a lower (above) and higher (below) magnification. **B.** Representative pictures of trichrome staining in samples with steatosis and HCC. In the latter both area with tumor and St/HCC were examined. Pictures showed the same area observed with a lower (above) and higher (below) magnification. Trichrome stains showed that collagen (green) was present only in portal space and, in limited amount, in perisinusoidal (Disse) spaces of lobule in both steatosis and St/HCC samples, in which macro and micro vesicular steatosis is also visible. Collagen was also present in correspondence of the capsule that delimits HCC (arrow). Bar: 100 µm.

**Figure 4:**
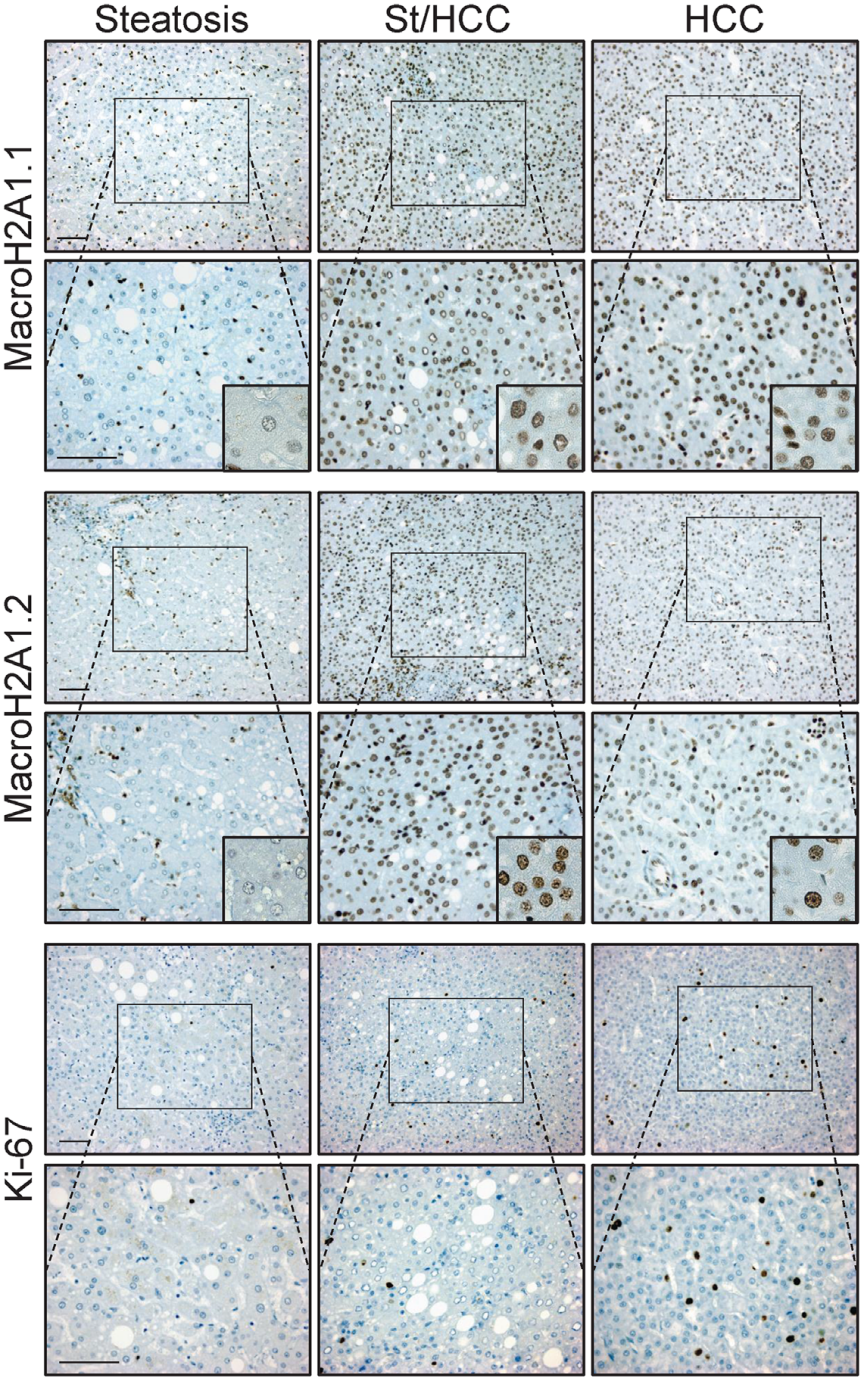
Representative pictures of immunostaining performed for MacroH2A1.1, MacroH2A1.2 and Ki-67 in samples with steatosis and HCC. In the latter both area with tumor and area of steatosis close to HCC (St/HCC) were examined. All nuclei of tumor cells were positive for either macroH2A1.1 or macroH2A1.2. Positivity in hepatocytes of steatosis was significantly lower. Pictures showed the same area observed with a lower (above) and higher (below) magnification. Insets show details of nuclear staining. Bar: 100 µm.

**Figure 5:**
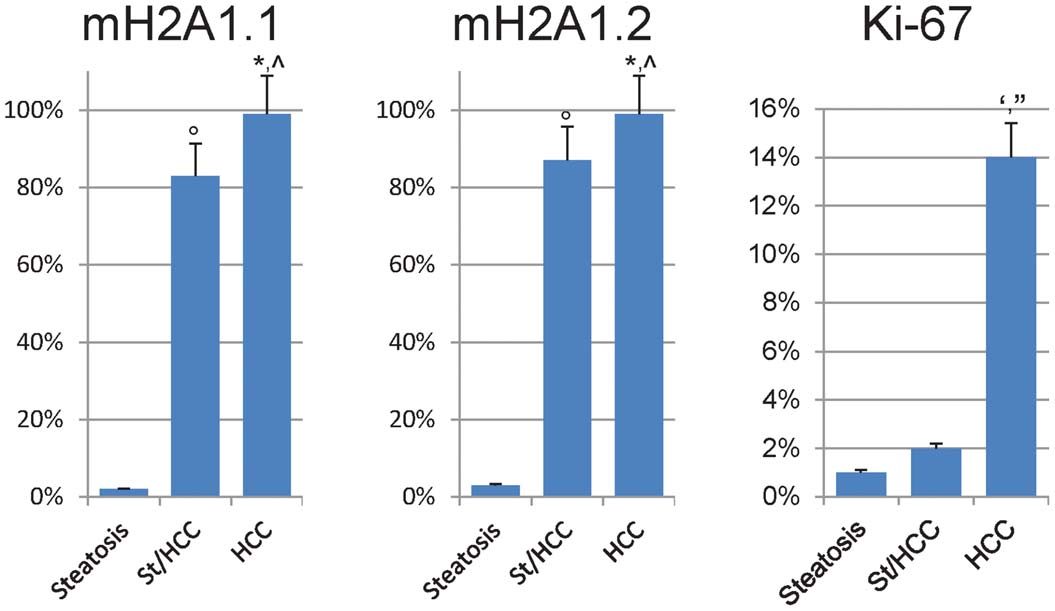
Histograms show statistical results for the evaluation of immunopositivity for macroH2A1.1, macroH2A1.2 and Ki-67 in samples of steatosis and HCC. In the latter both area with tumor and area of steatosis close to HCC (St/HCC) were examined. Significant differences (p<0.005) in the percentage of positive nuclei were found for both MacroH2A1.1 and MacroH2A1.2 between St/HCC and steatosis (°), HCC and steatosis (*) and HCC and St/HCC (∧). Significant differences (p<0.005) were also present for Ki-67 between HCC and steatosis (‘) and HCC and St/HCC (”).

Experiments were performed both in human samples with steatosis and samples with HCC (10 cases per each condition, Table 1). In the latter, areas with tumor and areas of steatosis close to HCC (St/HCC) were examined (Fig. 3A, Fig. 3B). Trichrome stain showed absence of fibrosis in all examined specimens. In particular, collagen was present only in portal space and, in limited amount, in perisinusoidal (Disse) spaces of lobule in both steatosis and St/HCC samples, as well as in the capsule that delimits HCC (Fig. 3B). Figure 4 shows representative immunostainings. Both macroH2A1.1 and macroH2A1.2 showed significant differences (p<0.005) in the percentage of positive nuclei between St/HCC and steatosis, HCC and steatosis and HCC and St/HCC (100% of tumor cells positive for either macroH2A1.1 or macroH2A1.2), when compared to steatosis (<2% of hepatocytes positive for macroH2A1.1 and macroH2A1.2) (Fig 4, Fig. 5). The St/HCC areas were highly immunopositive for macroH2A1.1 and macroH2A1.2, displaying 83% versus 88% of positive nuclei, respectively (p<0.05) (Fig 4, Fig. 5). Moreover, significant differences were also present for Ki-67 between HCC and steatosis and HCC and St/HCC (Fig. 4, Fig. 5). Finally, a number of nuclei in elements resembling to endothelial (sinusoidal) and perisinusoidal cells were also found positive both in steatosis and St/HCC areas (Fig. 4).

## Discussion

In this study we report that the histone variant macroH2A1 and its two splicing isoforms are strong markers of NAFLD-associated HCC, pointing to the importance of an epigenetic component in pathogenesis. One of the most striking epigenetic alterations that occur at the level of the chromatin is the exchange of the canonical H2A histone for histone variant macroH2A1, described nearly 20 years ago [32]. MacroH2A1 can play either a positive or negative role in transcriptional regulation in a context-dependent manner, and it can control cell cycle and proliferation [33]. The two exon splicing variants of macroH2A1, macroH2A1.1 and macroH2A1.2, differ by just 3 aminoacids and differentially bind NAD metabolites [34]. As referred to earlier, KO mice for both macroH2A1 isoforms display insulin resistance, hepatic steatosis and an altered expression of hepatic genes involved in lipid metabolism (lipoprotein lipase, CD36 and others) [16], [17], and alterations in the expression of macroH2A1.1 and macroH2A1.2 isoforms are associated to the occurrence/survival and/or the pathogenesis of various human cancers (lung, colon, melanoma) [19], [20], [22]. Our data show that both macroH2A1.1 and macroH2A1.2 protein expression levels are impressively increased in tumour tissue of human subjects presenting with HCC on a steatotic background without cirrhosis or fibrosis. The 10 patients studied with steatosis alone or with HCC show homogeneous and strongly consistent results. Immunohistochemistry analyses with specific antibodies showed that 100% of HCC nuclei were positive for macroH2A1 isoforms compared to surrounding liver parenchyma or to the liver of steatotic subjects without HCC. This was observed also in the liver of HCC mice models, either dietary/carcinogenic (HF/DEN) or genetic (PTEN KO), where HCC develops on the basis of a pre-existent NAFLD [8], [27]. Independently of the causes underlying steatosis, an increase in macroH2A1.1 and macroH2A1.2 is strongly associated to HCC development in these experimental models. mRNA levels for macroH2A1.1 and macroH2A1.2 in the animal models and in liver biopsies from patients were variable and did not reflect the differences observed in the protein levels found in steatosis and HCC (*data not shown*). This is consistent with a previous study, indicating that, differently from most tissues analyzed to date, in the liver the mRNA splicing that generate the two isoforms of macroH2A1 is not mirrored by changes in the protein levels [21]. We also found an interesting discrepancy between the NAFLD of the mouse models and that of the patients studied. In mice macroH2A1.2 expression is significantly increased in steatosis, whereas macroH2A1.1 is not; in human liver, mild content of fat alone was not associated to an increase of the isoforms (<2% of positive nuclei for either isoform). However, in the steatotic areas of the liver proximal to the HCC tissue, high immunopositivity for both macroH2A1.1 and macroH2A1.2 was present, with a slightly greater number of positive hepatocytes for macroH2A1.2 (88%) *versus* macroH2A1.1 (83%). This would be consistent with an involvement of macroH2A1.1 and/or macroH2A1.2 in the pathogenic progression of steatotic liver to malignant HCC in man.

One limitation of the mouse studies is that the antibodies used for detection of the macroH2A1 isoforms cannot be used in immunohistochemistry in this species. Consequently, immunoblotting data alone cannot distinguish between variations in macroH2A1.2 expression in the total liver histone extracts, if these are dependent from increases in hepatocyte expression or from other cell types, as non-hepatocyte cell types are stained in human liver immunohistochemistry (Fig. 4). Differences between HCC mouse models and HCC patients are multiple: mice do not develop HCC spontaneously, hence we manoeuvred to induce the disease either using an injection of DEN combined with a high fat diet, or by liver specific ablation of tumour suppressor PTEN, as previously described [8], [27]. There are also conflicting issues about the role of genes involved in hepatocarcinogenesis (i.e., MET, NF-κB, Stat3, Jnk, Shp2, and β-catenin) modelled in mice, which have arisen recently [35]. In any case, an increase of macroH2A1.2 observed only during fat accumulation may have metabolic implications. In this respect, the property of macroH2A1.1 in binding with very tight affinity NAD-derived metabolites, differently from macroH2A1.2 [34], is intriguing. NAD-derived metabolites such as O-acetyl-ADP ribose (OAADPR) is generated by the enzymatic reaction catalyzed by SIRT1, a NAD-dependent protein deacetylase, whose activation is considered protective against cellular metabolic and oxidative stresses, and against aging [36], [37]. Of note, liver-specific SIRT1 transgenic mice are protected against metabolic syndrome-associated HCC [38]. MacroH2A1.1 apparently suppresses growth of lung cancer cells and adenocarcinoma cells in a manner dependent on its ability to bind NAD-derived metabolites [21]. The presence of a metabolite-binding function in a chromatin component opens new potential connections between gene expression and lipid metabolism in the liver. Macro domains could also represent a novel tool for studying NAD metabolites and may be an attractive drug target [39].
