# Correction: Immunopositivity for Histone MacroH2A1 Isoforms Marks Steatosis-Associated Hepatocellular Carcinoma

**DOI:** 10.1371/annotation/b456329c-02fa-4055-afb8-2090cec17da6

**Published:** 2013-03-22

**Authors:** Francesca Rappa, Azzura Greco, Christine Podrini, Francesco Cappello, Michelangelo Foti, Lucie Bourgoin, Marion Peyrou, Arianna Marino, Nunzia Scibetta, Roger Williams, Gianluigi Mazzoccoli, Massimo Federici, Valerio Pazienza, Manlio Vinciguerra

There was information omitted from the Funding statement.

The correct statement is: This work was funded by the Italian Ministry of Health (GR program 2010-2311017) and the Associazione Italiana per la Ricerca sul Cancro (AIRC) program MyFAG. The funders had no role in study design, data collection and analysis, decision to publish, or preparation of the manuscript. 

